# From audit to policy: ESGE performance measures in centralized device-assisted enteroscopy services

**DOI:** 10.1055/a-2866-5739

**Published:** 2026-05-29

**Authors:** Gabriele Wurm Johansson, Anastasios Koulaouzidis, Ervin Toth

**Affiliations:** 1 Department of Gastroenterology 59564 Skåne University Hospital LundLundSkåne CountySweden; 2 Department of Clinical Research 6174 SDUOdenseRegion SyddanmarkDenmark; 3 Department of Surgery, SATC-C 11286 Odense UniversitetshospitalOdense/SvendborgRegion SyddanmarkDenmark; 4 Department of Gastroenterology 37805 Pomeranian Medical University in SzczecinSzczecinWest Pomeranian VoivodeshipPoland



Dear Editor,

The national Irish audit of device-assisted enteroscopy (DAE) by Costigan et al. 1 is a substantial, long-term service evaluation. The authors are to be congratulated for this large real-world dataset, benchmarked against European Society of Gastrointestinal Endoscopy (ESGE) performance measures 2 , that demostrates high safety standards, therapeutic success, and patient comfort are achievable in routine practice. The audit confirms that excellent outcomes are feasible within a centralized, high-volume model. However, extrapolating these findings to broader policy conclusions warrants caution, particularly given growing interest in using key performance indicators (KPIs) to guide service configuration. Although high-quality performance is shown in Ireland’s two DAE centers, the study does not establish centralization as the determinant of KPI attainment because both centers are high-volume referral units. In the absence of a national low-volume comparator, claims that centralization “ensures compliance” with ESGE KPIs remain conjectural. Comparisons with external cohorts, such as UK data 3 , are ecological and hypothesis-generating only, and risk reinterpreting KPIs as structural mandates rather than quality-improvement tools.

Furthermore, the KPI results themselves warrant refined interpretation. Several ESGE KPIs were not fully met, including appropriate indication, tattooing of maximal insertion depth, and lesion marking. This does not detract from service quality but reflects the purpose of ESGE KPIs: to support iterative audit and improvement rather than binary pass/fail judgments. Framing partial KPI attainment as evidence that a specific service configuration “achieves performance targets” risks oversimplifying both the intent and application of these measures. ESGE guidance emphasizes flexibility and contextual application, without proposing centralization as quality prerequisite. Using KPI compliance to implicitly advocate for national service reconfiguration, therefore, extends beyond the scope of the guidance and risks setting a precedent that may not be appropriate or transferable across healthcare systems.

The authors’ findings on sedation practice are important but should be interpreted in local context. Demonstrating that predominantly conscious sedation can achieve acceptable comfort and procedural effectiveness is valuable, particularly in resource-constrained systems. However, sedation choice in this cohort is shaped by structural availability and patient selection, limiting generalizability. ESGE guidance 4 supports conscious sedation, deep sedation, and general anesthesia based on procedural complexity and patient factors, underscoring that quality is compatible with multiple sedation strategies rather than a single preferred model.The discussion of “appropriate indication,” particularly in Crohn’s disease, raises a broader guideline-level issue. Procedures performed outside current ESGE indications may reflect evolving practice, but failure to meet a KPI threshold should not itself be interpreted as evidence that the KPI requires expansion. ESGE guidelines continue to recommend small-bowel capsule endoscopy as first-line investigation in many Crohn’s scenarios 5 , with DAE reserved for confirmation and therapy. Any redefinition of DAE indications would require broader evidence and formal guideline revision rather than post hoc reinterpretation of audit data.

In summary, Costigan et al. demonstrate that high-quality DAE can be delivered in a centralized, high-volume system. A more conservative interpretation frames the findings as evidence of feasibility rather than necessity. Preserving ESGE KPIs as quality-improvement tools, rather than policy levers for reorganization, will help promote excellence without unintended consequences for service diversity or access.
